# Binding of the transcription factor Atf1 to promoters serves as a barrier to phase nucleosome arrays and avoid cryptic transcription

**DOI:** 10.1093/nar/gku704

**Published:** 2014-08-13

**Authors:** Patricia García, Esther Paulo, Jun Gao, Wayne P. Wahls, José Ayté, Ernesto Lowy, Elena Hidalgo

**Affiliations:** 1 Oxidative Stress and Cell Cycle Group, Departament de Ciències Experimentals i de la Salut, Universitat Pompeu Fabra, C/ Dr Aiguader 88, 08003 Barcelona, Spain; 2 Department of Biochemistry and Molecular Biology, University of Arkansas for Medical Sciences, 4301 W Markham St., Little Rock, AR 72205, USA; 3 Core Facilities, Centre for Genomic Regulation, Universitat Pompeu Fabra, C/ Dr Aiguader 88, 0800 Barcelona, Spain

## Abstract

*Schizosaccharomyces pombe* displays a large transcriptional response common to several stress conditions, regulated primarily by the transcription factor Atf1. Atf1-dependent promoters contain especially broad nucleosome depleted regions (NDRs) prior to stress imposition. We show here that basal binding of Atf1 to these promoters competes with histones to create wider NDRs at stress genes. Moreover, deletion of *atf1* results in nucleosome disorganization specifically at stress coding regions and derepresses antisense transcription. Our data indicate that the transcription factor binding to promoters acts as an effective barrier to fix the +1 nucleosome and phase downstream nucleosome arrays to prevent cryptic transcription.

## INTRODUCTION

Cells have the capacity to adapt to external conditions and to induce massive changes on their gene expression patterns to allow survival. In *Schizosaccharomyces pombe*, the MAP kinase Sty1 and the basic leucine zipper (bZIP) transcription factor (TF) Atf1 regulate up to 400 genes in response to several stress conditions (1,2). Atf1 bound to a cyclic AMP response element (CRE) DNA site in promoters controls stress-induced chromatin remodelling and transcription (3). Recently, our group reported that these high plasticity stress genes, whose expression significantly change upon environmental stresses, display an especially wide and deep nucleosome depleted region (NDR) upstream of the transcription start site (TSS) even prior to stress imposition (1). Transcriptional activation of these genes does not alter promoter nucleosome occupancy, but induces eviction of downstream nucleosomes at coding regions. The histone acetyl-transferase Gcn5 mediates such nucleosome eviction after stress treatment, allowing efficient RNA polymerase II progression and transcription (1). A key question is what factors establish NDRs in promoters of Atf1-dependent genes under basal conditions.

In several organisms genome-wide nucleosome maps show a common nucleosome organization at promoters, with a region relatively free of nucleosomes upstream the TSS bordered by two arrays of well-positioned nucleosomes with decaying occupancy and regularity along the gene (4,5). DNA sequence contributes to some extent to generate such NDRs, e.g. poly(dA-dT) tracts found at budding yeast promoters disfavour strong bending of DNA and in turn exclude nucleosomes favouring NDRs (6). However, DNA sequence is clearly not the only element involved in nucleosome positioning. Some *trans* factors may also be involved in NDR formation: several *in vivo* evidences suggest that yeast TFs may compete with histones at promoters to generate NDRs (7–9). Furthermore, it has been described that the addition of ATP to yeast whole-cell extracts is required for proper *in vitro* reconstitution of nucleosomes around the TSS (10).

Fission yeast promoters do not seem to be enriched in poly(dA-dT) tracts (11,12). How, then, are the NDRs established? Since Atf1 is bound to its specific CRE site at promoters even at basal conditions (13) and remodels chromatin structure (3), we tested whether this TF plays a role in NDR establishment. We used nucleosome scanning assays to study the nucleosome pattern at some specific stress promoters, as well as nucleosome sequencing for genome-wide nucleosome patterning in the presence and absence of Atf1. We found that disruption of Atf1-to-DNA binding by deletion of the TF or by elimination of the DNA binding sites at stress promoters resulted in narrower NDRs. We used genome-wide sequencing to show that, unexpectedly, Atf1 was important for maintaining the nucleosome arrays along coding regions of stress genes, and to silence production of antisense transcripts. These results suggest that Atf1 mediates NDR establishment and may function as an effective barrier for proper downstream nucleosome positioning, preventing cryptic transcription.

## MATERIALS AND METHODS

### Plasmid construction

The p428 and p428.bZIP integrative plasmids encode the HA-tagged Atf1 full-length protein (566 amino acids) or only the C-terminal region of Atf1 containing the bZIP domain respectively (from 396 to 566 amino acids); the chimeric genes are under the control of the constitutive *sty1* promoter. Briefly, the full length or truncated *atf1* coding sequences were polymerase chain reaction (PCR)-amplified from an *S. pombe* cDNA library using specific primers. Each PCR product was digested with BglII/SmaI and cloned into the plasmid p386 (14) that contains the constitutive *sty1* promoter (0.8 kb from ATG) fused to the HA coding sequence, digested with BamHI/SmaI. The p413 integrative plasmid encodes the DNA-binding domain of budding yeast Gal4 (residues 1–147; Gal4 DNA binding domain) and a FLAG epitope (DYKDDDDK) (15). The Gal4–FLAG region was PCR-amplified from the plasmid pSP1 (15,16), digested with BglII–BamHI and cloned into the plasmid p386 described above digested with BamHI. Similar strategy was followed to construct the integrative plasmid p411.ΔbZIP that encodes the chimeric protein Gal4DBD–FLAG–HA fused to the N-terminal region of Atf1, containing residues 1–395 which lacks the C-terminal bZIP of Atf1. Each fusion protein was under the control of the constitutive *sty1* promoter. The integrity of each of these constructs was confirmed by DNA sequencing.

### Yeast strains and growth conditions

We used the wild-type (WT) *S. pombe* strain 972 (*h^−^*) and 975 (*h^+^*) and mutants thereof. The origins and genotypes of strains in this study are indicated in Supplementary Table S1. Strains EP203 and EP203.bZIP were constructed integrating the plasmid p428 and p428.bZIP respectively in the EP193 strain at *leu1^+^* locus. To construct strains EP184 and EP255 with CRE-to-G4BS substitutions, we first deleted 160 bp of the *ctt1* or *gpd1* promoters in the strain CH1364 (*ura5-294 lys7-2 leu1-32*) with a cassette containing both the *ura5^+^* and the *lys7^+^* genes, as recently described by the group of Hoffman (17). Then, we replaced such genes by recombination with a linear fragment of 500 bp of the *gpd1* or *ctt1* promoters with the CRE site changed by a Gal4 binding site, and selected the uracil auxotrophic clones by resistance to 5-fluoroorotic acid (5-FOA; Toronto Research Chemicals Inc.); we double checked lysine auxotrophy in the selected clones. The resulting strains were crossed out to wild-type strains to eliminate auxotrophies, yielding strains EP184 and EP255. In EP184, the CRE site (ATGACGT) at *ctt1* promoter was replaced by a Gal4 binding site by substitution of −435 to −419 from the translational start site by the following G4BS: CGGAAGACTCTCCTCCG. In the EP255 strain, the CRE site (TTACGTCA) at *gpd1* promoter was replaced by substitution of −329 to −312 from the translational start site by the G4BS. Strains EP213 and EP286 were constructed by integrating the plasmid p413 at the *leu1^+^* loci of strains EP184 and EP255, respectively. Strains EP212.ΔbZIP and EP287.ΔbZIP were constructed by integrating the plasmid p411.ΔbZIP at the *leu1^+^* loci of strains EP184 and EP255, respectively. Cells were grown in liquid- or solid-rich medium (YE5S) or in synthetic minimal medium (MM) as described previously (18). When indicated, 35 μg/ml of trichostatin A (TSA; Sigma) was added to rich media cultures with an OD_600_ of 0.03, and growth proceeded for ∼2 days to reach an OD_600_ of 7–8 (eight generations). Cultures were then centrifuged, cell pellets washed from TSA, resuspended in rich media, and growth proceeded performing one culture dilution with rich media, to reach in ∼20 h an OD_600_ of 0.5 after eight doublings. For survival on solid plates, cells before, during or after TSA withdrawal were serial diluted and spotted on YE5S plates containing or not 1 mg/ml of 5-FOA, or on minimal media plates lacking uracil.

### Nucleosome-scanning analysis

Mononucleosomes were obtained as described before (1,11,19), and the resulting DNA was analysed by qPCR as described previously (1,20). Briefly, strains were cultured in 250 ml of YE5S medium to an OD_600_ of 0.5 and then cross-linked with formaldehyde (final concentration of 0.5% (v/v)) for 20 min at 25°C. Cells were then digested with Zymolyase 20T (Amsbio), and spheroplasts were treated with increasing concentrations of micrococcal nuclease (MNase; Sigma). Purified DNA was separated electrophoretically, and samples displaying 80–90% mononucleosomal DNA without subnucleosomal fragments (faster migration than mononucleosomes in the electrophoresis) were further analysed by qPCR with a set of overlapping primer pairs (see Supplementary Table S2). For each primer pair, numbers in Y-axis correspond to the relative value to the input, which was obtained using as a template DNA from cells not treated with MNase, and received a value of 1.

### Nucleosomal DNA preparation, sequencing and data processing

Fixed cells were subjected to chromatin isolation and MNase digestion as described above for nucleosome-scanning analysis (1,19). Again, the MNase concentration has to be carefully optimized to discard samples with underdigested or overdigested chromatin (we only purified mononucleosomes from samples with 80–90% of mononucleosomal fraction), since the extent of the digestion has to be very similar to compare nucleosome maps for different strains or experiments (21). For each nucleosomal map to be determined, mononucleosomal DNA fragments from two independent cell cultures were purified from 2% agarose gels run in Tris-acetic acid-EDTA (TAE), DNA was extracted with Quantum Prep Freeze'N Squeeze DNA gel extraction spin columns (Bio-Rad), pooled and subjected to single-end sequencing using Illumina platform. Only one ultrasequencing reaction was performed for nucleosomes of wild-type and *Δatf1* cells expressing HA-bZIP, while two full biological replicates (including duplicate sequencing reactions) were performed for *Δatf1* nucleosomes. Short reads produced in this work (26–50 millions of which 98% were uniquely mapped) were aligned against the genome of *S. pombe* 972 using the Bowtie2 software (22) with default parameters. The SAM format file produced by Bowtie2 was converted into a BAM file using SAMtools (23) and then the Bioconductor nucleR package (24) was used to process the alignment file and to remove noise from the genomic data. First, we estimated the average insert size of each sequencing library using the fragmentLenDetect nucleR's method. Then, the single end reads were processed using the processReads nucleR's method by setting its trim argument to 40. This method will shift the start coordinate of each read by half the estimated fragment size, and will use this new shifted coordinate to extend 20 nucleotides on each side to produce the final start and end coordinates. In this way, we are sure that we are analyzing the nucleosome dyad. nucleR was also used to normalize the coverage values by dividing each value by the total number of reads mapped and then multiplying per one million. Finally, the nucleR's filterFFT method was used to smooth the genomic data and to reduce the noise. This processed data was exported as a WIG format file (http://genome.ucsc.edu/goldenPath/help/wiggle.html) and then we developed an in-house R [R Core Team (2013)] script to produce a data matrix in which each row is the genomic coordinate of the +1 nucleosome for the selected genes and each column is the nucleR's processed signal value for a certain genomic coordinate located in the region spanning ±500 nucleotides around the +1 nucleosome.

### RNA analysis

Total RNA from cultures was obtained, processed and transferred to membranes as described previously (7). Membranes were hybridized with the [α-^32^P]dCTP-labelled *gpd1*, *hsp9, ctt1, sty1* or *act1* probes.

### Preparation of protein extracts and immunoblot analysis

To analyse the amount of Atf1, trichloroacetic acid extracts were prepared as previously described (13). Immunoblotting was performed using monoclonal anti-HA (12CA5) and polyclonal anti-Atf1 antibodies (13). Anti-Sty1 (25) antiserum was used as a loading control. The blots were quantified using the ImageJ program, using the constitutive Sty1 levels as loading controls. Numbers show the fold-induction relative to the wild-type strain.

### Chromatin immunoprecipitation

The indicated strains were grown in minimal media, and chromatin isolation and immunoprecipitation was performed as described previously (1).

### Strand-specific reverse transcription-qPCR

RNA was purified, treated with DNAse and subjected to reverse transcription (Reverse Transcription System, Promega) using primers complementary to either forward or reverse transcripts. Reverse-transcribed cDNAs were amplified by real-time PCR using gene-specific primers (Supplementary Table S3).

## RESULTS

### Atf1 mediates NDR formation at stress genes

**Figure 1.**
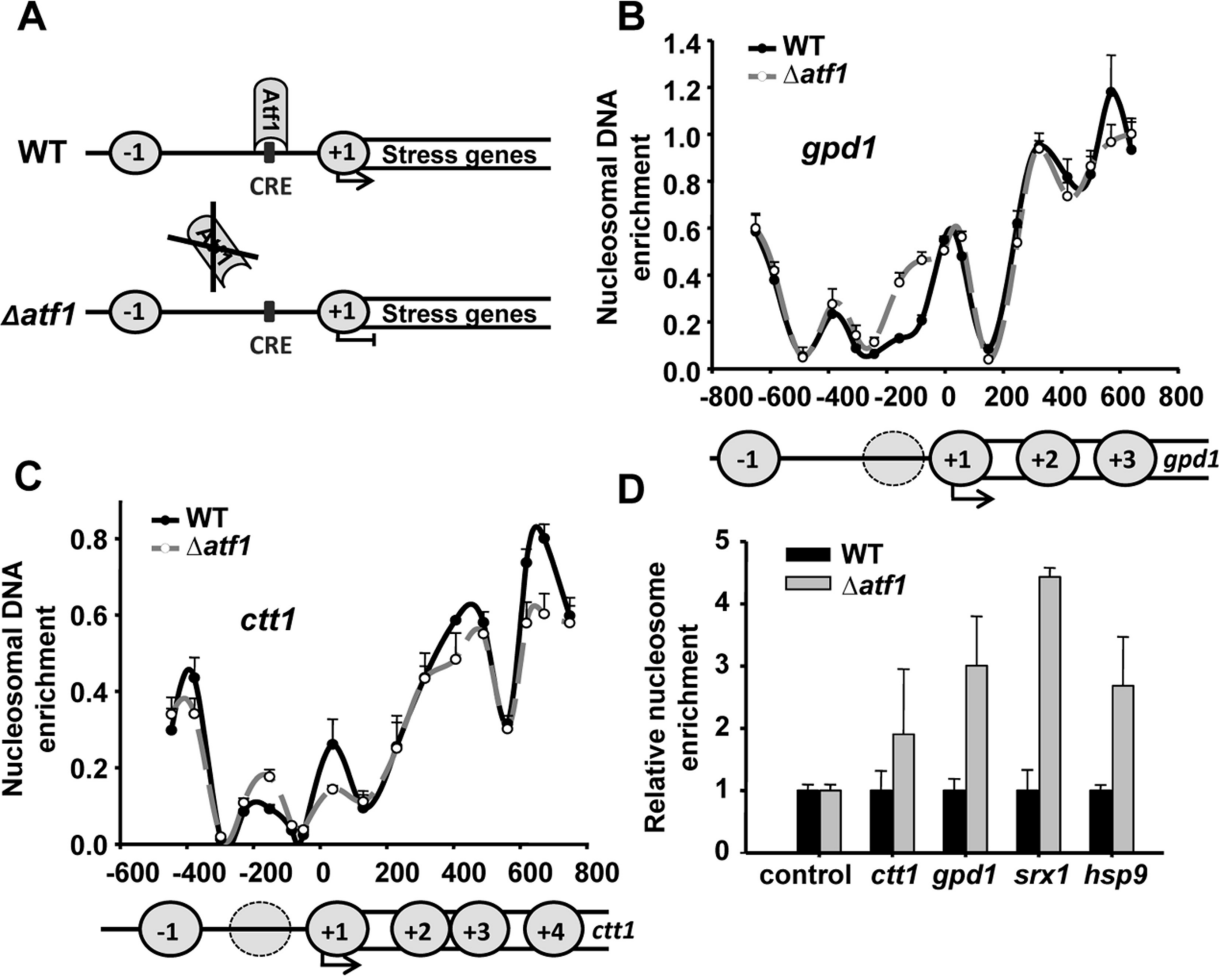
Atf1promotes NDR formation at stress genes. (**A**) Schematic representation of the Atf1 DNA-binding at the CRE site of stress promoters in wild-type and Δ*atf1* strains. (B and C) Mononucleosomes were isolated from cultures of strains 972 (WT) and MS98 (Δ*atf1*). qPCR was performed using 17 pairs of primers covering 1.3 and 1.2 kb of the promoters, TSS (black arrow) and coding regions (white rectangle) of *gpd1* (**B**) or *ctt1* (**C**), respectively. The values of nucleosome occupancy for each qPCR reaction (Y-axis) is plotted against the gene position of the centre of the PCR-amplified fragment for each primers pair, relative to the TSS, with a value of 0 (X-axis). Nucleosomes are represented as circles. (**D**) Relative nucleosome occupancy was determined as described above using primers for *mei2* (control), *ctt1*, *gpd1*, *srx1* and *hsp9* promoters. The graph shows the nucleosome occupancy at Δ*atf1* cells relative to wild-type strain, with an assigned value of 1 in each gene. Error bars (SEM) were calculated from biological triplicates.

To test whether Atf1 is required for chromatin organization at stress promoters, we determined the position of nucleosomes at two Atf1-dependent genes (*gpd1* and *ctt1*) by nucleosome scanning. Briefly, we treated chromatin from wild-type and Δ*atf1* strains (Figure 1A) with MNase, isolated mononucleosomes and PCR-amplified them with pairs of overlapping primers covering promoters and 5′-end of coding sequences of the *gpd1* and *ctt1* genes, as described elsewhere (1). We confirmed the presence of wide NDRs upstream of the TSS of both genes, as previously described (1,19). However, Δ*atf1* cells displayed higher relative nucleosome occupancy exactly where the CRE sites are (Figure 1B and C). Next, we determined the nucleosome occupancy with primers close to the CRE site of two additional Atf1-dependent genes, *srx1* and *hsp9* (Figure 1D), confirming increased nucleosome occupancy in cells lacking Atf1. These results indicate that Atf1 may compete with histone binding at stress promoters and decrease nucleosome occupancy favouring NDR formation.

### DNA-binding of the Atf1 bZIP domain alone is enough for NDR establishment

**Figure 2.**
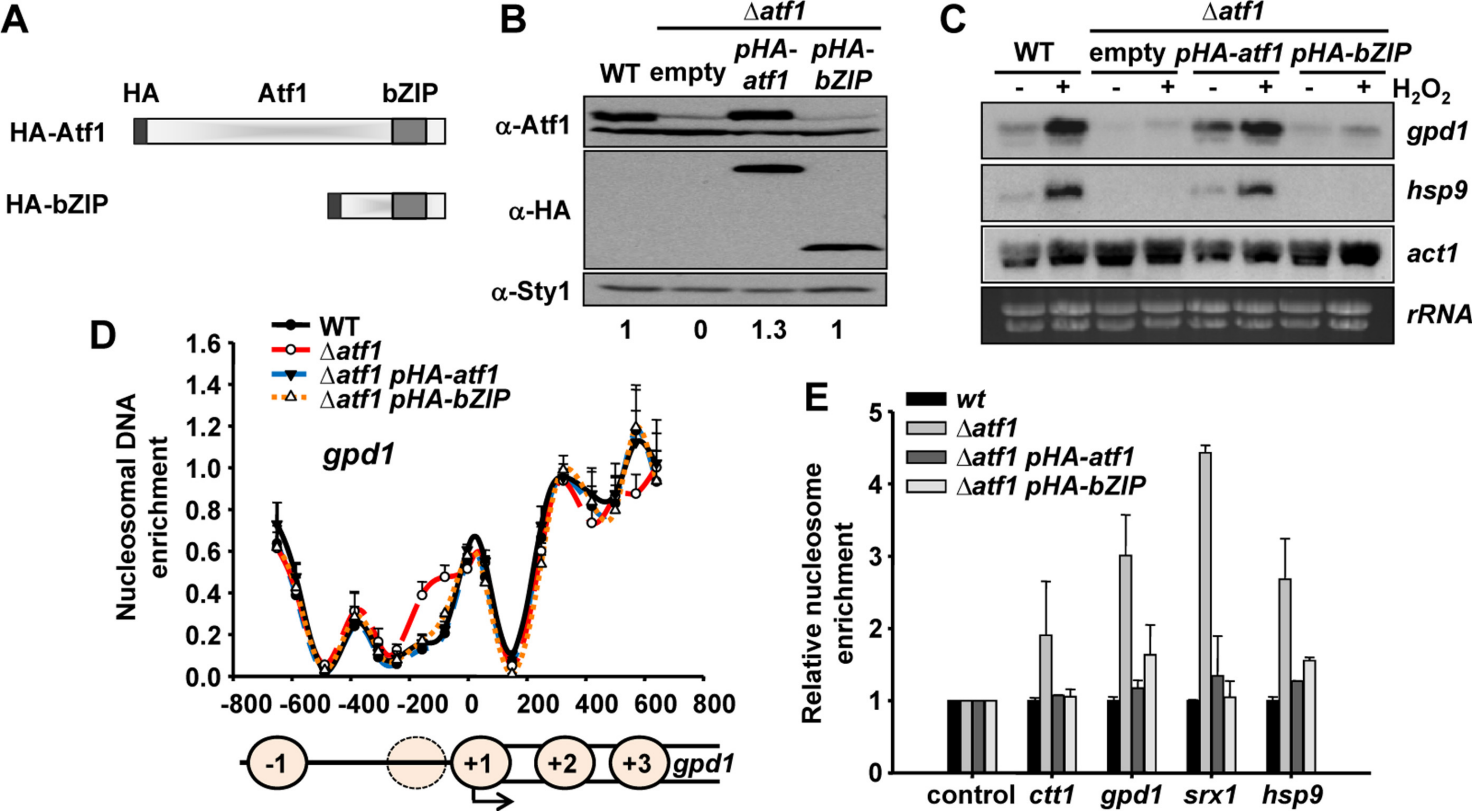
DNA-binding of Atf1 or the Atf1 bZIP domain alone is sufficient to restore the NDR in Atf1-deficient cells. (**A**) Schematic representation of HA-Atf1 and HA-bZIP proteins. (**B**) Protein extracts from strains 972 (WT), MS98 (Δ*atf1*), EP203 (Δ*atf1 pHA-atf1*) or EP203.bZIP (Δ*atf1 pHA-bZIP*) were analysed by western blot with antibodies against Atf1 (amino part), HA or Sty1, as a loading control. Relative units indicate the fold-differences of Atf1 levels in the different strains relative to WT, with an assigned value of 1 (**C**) Stress-dependent transcriptional analysis of strains as in (B), treated or not with 1 mM H_2_O_2_ for 15 min. Total RNA was analysed by northern blot with probes for *gpd1*, *hsp9* and *act1*. *rRNA* and *act1* are shown as loading controls. (**D**) Mononucleosomes were isolated and nucleosome scanning performed as described in Figure 1B for the strains as in (B). (**E**) Relative nucleosome occupancy was determined as described in Figure 1D for the strains as in (B).

Atf1 could participate in NDR formation by either a direct competition with histones at promoters, or by playing an indirect role recruiting other proteins, e.g. RNA polymerase II, chromatin remodellers or histone modifiers. In order to distinguish between both possibilities we expressed in Δ*atf1* cells either a full length or a truncated Atf1 protein, containing only the bZIP domain (Figure 2A). Both proteins were expressed to levels similar to those of wild-type cells (Figure 2B). As expected, full-length Atf1 was completely functional and restored the H_2_O_2_-dependent transcriptional response to Δ*atf1* cells, whereas cells expressing HA-bZIP failed to respond to stress (Figure 2C). Interestingly, cells expressing either full-length Atf1 or only its bZIP domain in a Δ*atf1* background recapitulated the wild-type nucleosome pattern (Figure 2D and E), indicating that binding of the bZIP domain alone is sufficient to maintain an opened chromatin structure and form a broad NDR. Even though we cannot fully rule out the recruitment of other proteins by the bZIP domain, this result suggests a direct role of Atf1 competing with histones at stress promoters.

### NDR formation is impaired at promoters lacking the Atf1-binding CRE site

**Figure 3.**
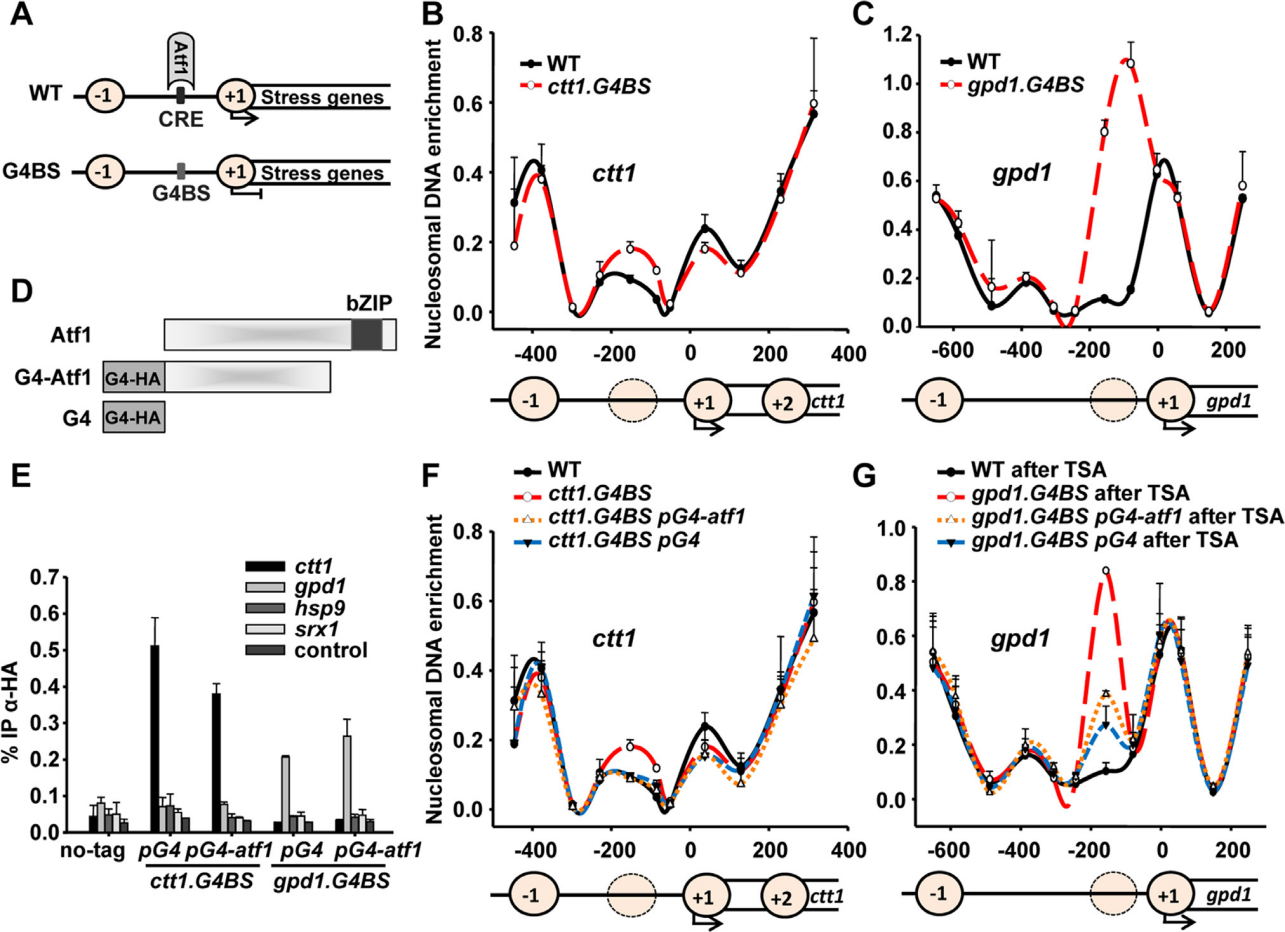
CRE site substitution by Gal4-binding site reduces NDR which is restored by artificially tethering Gal4-Atf1 or the Gal4 binding domain. (**A**) Schematic representation of CRE site substitution by a Gal4 binding site (G4BS). (B and C) Nucleosome-scanning analysis was performed as described in Figure 1B and C from cultures of strains 972 (WT) and EP184 (*ctt1*.*G4BS*; **B**) or EP255 (*gpd1*.*G4BS*; **C**). (**D**) Schematic representation of the fusion proteins Gal4-Atf1^1–395^ (lacking the bZIP; G4-Atf1) and Gal4 DNA binding domain (G4). (**E**) Gal4 binding domain and Gal4-Atf1^1–395^ physically bind to *ctt1* or *gpd1* promoters with a CRE-to-G4BS substitution. Cultures of strains 972 (no-tag), EP213 (*ctt1*.*G4BS pG4*), EP212.ΔbZIP (*ctt1*.*G4BS pG4-atf1*), EP286 (*gpd1*.*G4BS pG4*) and EP287.ΔbZIP (*gpd1*.*G4BS pG4-atf1*) were used to perform ChIP experiments using primers at *ctt1*, *gpd1*, *hsp9* and *srx1* promoters. An intergenic region was used as a negative control (control). Error bars (SEM) for all ChIP experiments were calculated from biological triplicates. (**F**) Mononucleosomes were isolated from cultures of strains 972 (WT), EP184 (*ctt1*.*G4BS*), EP213 (*ctt1*.*G4BS pG4*) and EP212.ΔbZIP (*ctt1*.*G4BS pG4-atf1*), and nucleosome scanning performed as described in Figure 1B. (**G**) Mononucleosomes were isolated from cultures of strains 972 (WT), EP255 (*gpd1*.*G4BS*), EP286 (*gpd1*.*G4BS pGal4*) and EP287.ΔbZIP (*gpd1*.*G4BS pG4-atf1*) treated with trichostatin A for eight generations, then washed and allowed to grow for eight generations without the drug; nucleosome scanning was then performed as described in Figure 1B. Error bars (SEM) were calculated from biological duplicates (B, C, F and G).

To further investigate the role of Atf1 in nucleosome organization we decided to replace the CRE site of *gpd1* and *ctt1* promoters with a Gal4 binding site (*gpd1*.*G4BS* and *ctt1*.*G4BS*) (Figure 3A). As shown in Figure 3B, *ctt1*.*G4BS* promoter showed higher nucleosome occupancy than wild-type cells just upstream of the TSS coinciding with the Gal4-binding site. The same was observed at *gpd1*.*G4BS* promoter although to a higher extent (Figure 3C). The nucleosome occupancy showed at both mutated promoters was even higher than in CRE-containing promoters of Δ*atf1* cells (Figure 1B versus Figure 3C and Figure 1C versus Figure 3B). CRE site elimination may have greater consequences than Atf1 deletion because other bZIP factors may bind, although less efficiently, to such a site in the absence of Atf1 (15).

The DNA-binding domain of the budding yeast Gal4 protein coupled to Atf1 has been previously used to tether the Atf1 TF to an engineered *ade6* gene (containing a Gal4 site) and promote hotspot meiotic recombination (15). We constructed two plasmids allowing the expression of HA-tagged Gal4 DNA binding domain alone (G4 in Figure 3D) or fused to Atf1^1–395^, only lacking the C-terminal bZIP domain (G4-Atf1 in Figure 3D). Elimination of the bZIP domain prevented G4-Atf1 binding to other stress promoters or to other less canonical CRE sites at *gpd1* and *ctt1*. We then expressed either G4 or G4-Atf1 proteins in cells with the CRE site of *gpd1* or *ctt1* promoters replaced by the Gal4 binding site. As expected, chromatin immunoprecipitation (ChIP) assay showed specific binding of both proteins (G4 and G4-Atf1) to *G4BS*-containing *gpd1* or *ctt1* promoters, but not to other stress promoters (Figure 3E).

To verify the role of Atf1 in generating NDRs at stress genes, we determined the nucleosome maps at the mutated promoters expressing either Gal4 binding domain alone or Gal4 binding domain fused to Atf1. Interestingly, the nucleosome maps in cells expressing either G4 or G4-Atf1 were similar to one another and from wild-type cells at *ctt1*.*G4BS* promoter (Figure 3F). This result reveals the ability of Atf1 and other DNA-binding domains (such as G4) to compete with histones and establish NDRs at stress promoters.

Regarding cells containing the *gpd1*.*G4BS* promoter, expression of G4 or G4-Atf1 caused only a slight decrease in nucleosome occupancy (Supplementary Figure S1A). Since the new nucleosome around the *G4BS* at *gpd1*.*G4BS* promoter seems to be strongly positioned (Figure 3C), the Gal4 DNA-binding domain may not be able to easily compete with histones. Accordingly, ChIP experiments suggested weaker occupancy of both proteins to *gpd1*.*G4BS* promoter than to *ctt1*.*G4BS* (Figure 3E). To favour TF-to-histones competition at the *gpd1*.*G4BS* promoter, we studied *de novo* nucleosome deposition by treating cells with a histone deacetylase inhibitor, TSA, which leads to hyperacetylation of histones H3 and H4 and may decrease the affinity of nucleosomes for DNA *in vivo* (26). Thus, we treated cells with TSA for eight generations to impair wrapping of DNA around nucleosomes, and then we washed the drug and allowed nucleosome repositioning for another eight generations (Supplementary Figure S1B). We checked TSA efficacy by treating wild-type and Δ*dcr1* cells mutated in the centromeric *otr1* locus. As previously reported (26), this treatment alleviated *otr1::ura4* silencing; after TSA withdrawal, wild-type (but not Δ*dcr1*) cells fully re-established silencing demonstrating *de novo* nucleosome deposition (Supplementary Figure S1C). As already reported for the *nmt1* and *fbp1* genes (26), TSA treatment altered expression of euchromatic genes: it derepressed expression of the *gpd1* and *ctt1* genes, and induced the expression of a shorter *sty1* mRNA (Supplementary Figure S1D; +TSA). However, after eight generations of TSA withdrawal, mRNA expression levels returned to normal (Supplementary Figure S1D; after TSA). Consistently, during TSA treatment our nucleosome scanning analysis failed to detect proper nucleosome positioning around the *gpd1* gene (Supplementary Figure S1E). As expected, the map of nucleosome positions at the *gpd1* gene was completely re-established after TSA withdrawal (compare wild-type map in Figure 3C and G). We determined using ChIP that binding of G4 and G4-Atf1 to *gpd1*.*G4BS* was stronger after TSA treatment than in untreated cells (Supplementary Figure S1F). This binding of G4 and G4-Atf1 precluded histones from binding to the *gpd1*.*G4BS* promoter: cells expressing G4 or G4-Atf1 proteins showed a dramatic decrease in nucleosome occupancy (Figure 3G). This result suggests an active competition between DNA-binding proteins and histones at the 5′ ends of genes.

### Atf1 promotes phased nucleosome array formation and prevents cryptic transcription

**Figure 4.**
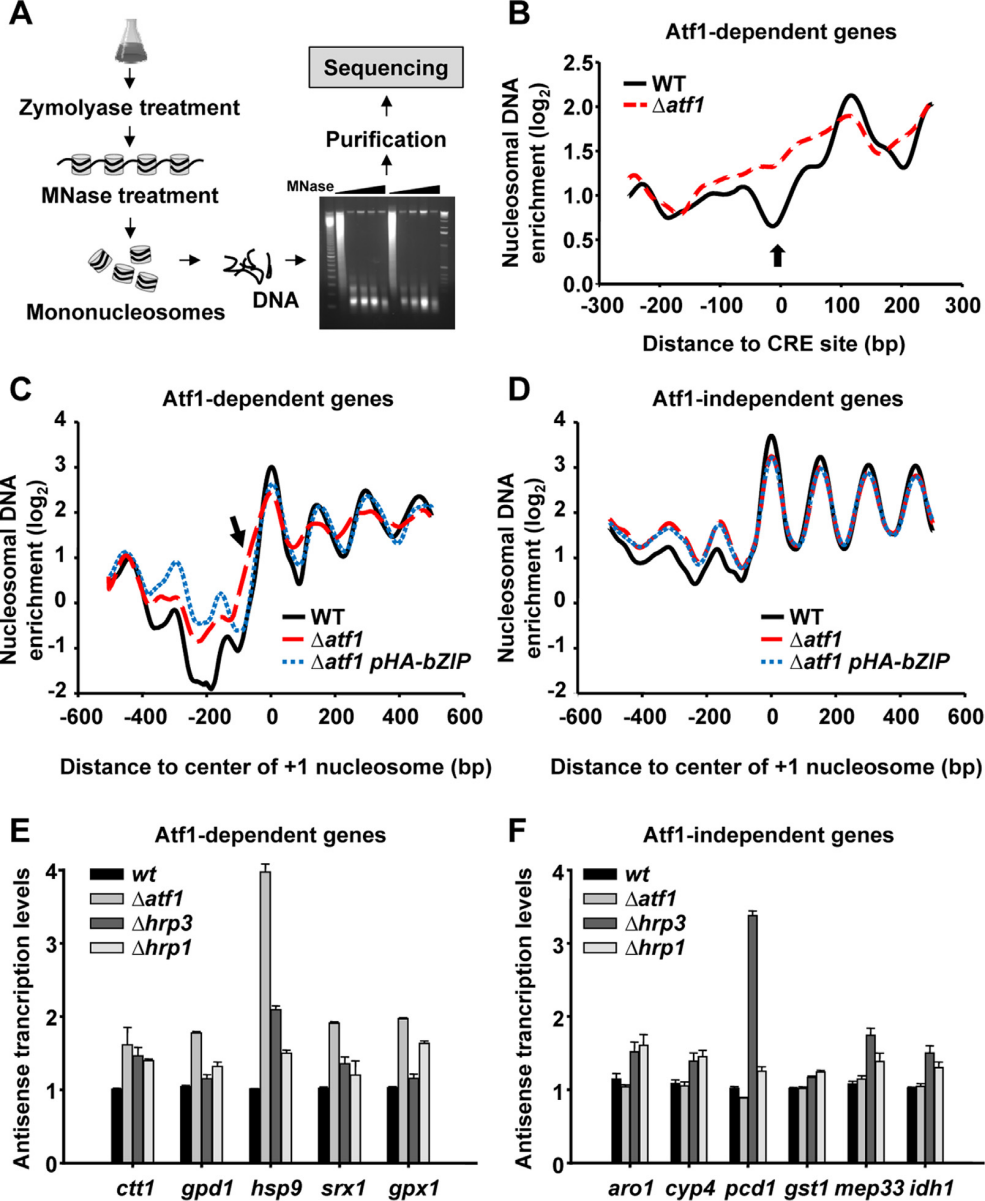
The regularly organized nucleosome arrays in stress coding regions are disrupted in the Δ*atf1* strain causing enhanced antisense transcription. (**A**) Scheme depicting the MNase-seq assay. (**B**) Composite plots of relative nucleosome occupancy for strains 972 (WT) and MS98 (Δ*atf1*). Thirty five stress genes were aligned at their CRE sites and the average of their log_2_ nucleosome occupancy was plotted. (**C**) Composite plots of relative nucleosome occupancy for strains 972 (WT), MS98 (Δ*atf1*) and EP203.bZIP (Δ*atf1 pHA-bZIP*). The genes represented in (B) were aligned at their +1 nucleosome and the average of their log_2_ nucleosome occupancy data was plotted. (**D**) Fifty Atf1-independent genes were aligned as in (C). (**E** and **F**) The expression levels of antisense transcripts in strains 972 (WT), MS98 (Δ*atf1*), EP110 (Δ*hrp3*) and IV69 (Δ*hrp1*) for Atf1-dependent (*ctt1*, *gpd1*, *hsp9*, *srx1* and *gpx1*; **E**) and -independent (*aro1*, *cyp4*, *pcd1*, *gst1*, *mep33* and *idh1*; **F**) genes were analysed by reverse transcription-qPCR. The graphs show the amount of transcripts of each gene relative to that in the wild-type strain. Error bars (SEM) were calculated from biological triplicates.

In order to pinpoint the defects on genome-wide nucleosome organization of *Δatf1* cells, we explored the global nucleosome occupancy profiles of wild-type and Δ*atf1* strains using MNase-seq (MNase digestion followed by sequencing). After mild cross-linking and MNase digestion, nucleosomal DNA fragments were isolated, purified from agarose gels and subjected to single-end sequencing using an Illumina platform (Figure 4A). The Bioconductor nucleR package (24) was used to remove noise and estimate nucleosome occupancy. Total reads in each sample were normalized using the unit RPM (reads per million) as described elsewhere (27).

We obtained a genome-wide nucleosome map with 90% overlap with that described by Shim *et al.*, also obtained by MNase-seq (27). We further compared our nucleosome sequencing data with a microarray-based nucleosome map report (11). Relative nucleosome positioning and NDRs from both datasets were comparable in the stress genes we analysed (Supplementary Figure S2; compare WT/our data with Shim *et al.*). We also applied to the *S. pombe* genome the program designed by Segal *et al.*, which is supposed to infer the position of nucleosomes based exclusively on DNA sequence (6); as observed in Supplementary Figure S2, this program did not reveal NDRs at stress promoters, confirming that factors other than base composition should direct nucleosome exclusion at these loci.

We used our genome-wide nucleosome maps to investigate the role of Atf1 in nucleosome exclusion determining the relative nucleosome occupancy at CRE sites of genes showing the highest induced expression under H_2_O_2_ stress, determined by microarray data (1,28) (Supplementary Table S4). The average nucleosome profile of these Atf1-dependent genes revealed a region depleted of nucleosomes coinciding with the CRE site that was not present in Δ*atf1* cells (Figure 4B). A very similar profile was observed when other 35 CRE-containing promoters, extracted from an Atf1-ChIP-sequencing report (Supplementary Table S5), were added to the analysis (Supplementary Figure S3A). This result correlated with nucleosome occupancy showed at *gpd1* and *ctt1* promoters in the absence of Atf1 (Figure 1B and C) and indicates a general situation in which Atf1 DNA-binding impairs nucleosome deposition.

It has been proposed that NDRs may potentially serve as barriers that, by locally preventing nucleosome establishment, may promote statistical positioning of flanking nucleosomes forming an array emanating from the barrier (for a review, see (29)). However, this pattern is not apparent *in vitro* and it has recently been demonstrated that this is not a passive process since ATP-dependent remodellers are required to create an array around the TSS (10,27,30,31). We decided to analyse the nucleosome positioning at stress genes in wild-type and *Δatf1* strains. Atf1-dependent genes (Supplementary Table S4) were aligned at their first nucleosome (+1) and the average of their log_2_ nucleosome occupancy data was plotted (Figure 4C). Wild-type cells showed the classical nucleosome organization pattern, with a pronounced NDR upstream of the TSS flanked by a highly positioned +1 nucleosome and an organized nucleosome array at coding regions (Figure 4C, solid line). This tightly positioned nucleosome array downstream of the TSS was also observed in data extracted from published data by Shim *et al.* (Supplementary Figure S3B, dashed line), even though the average NDR at stress genes was less pronounced in this wild-type dataset. Interestingly, *Δatf1* cells showed a wider +1 nucleosome position with a shoulder getting into the NDR, indicating a defect defining the position of the first nucleosome (Figure 4C, arrow, large dashed line, and Supplementary Figure S3C). As a result, the NDR becomes narrower, in agreement with the nucleosome patterns showed at *gpd1* and *ctt1* promoters (Figure 1B and C). Furthermore, the phased nucleosome array downstream of +1 quickly vanishes in cells lacking Atf1 (Figure 4C and Supplementary Figure S3C). As a control, we selected 50 Atf1-independent genes (Supplementary Table S6) (28), and analysed their nucleosome arrays: wild-type and *Δatf1* cells shared the same nucleosome profile along the coding regions (Figure 4D and Supplementary Figure S3D), indicating that the nucleosome disorganization is specifically observed in genes regulated by Atf1. Importantly, expression of only the bZIP domain of Atf1 in *Δatf1* cells restored the phased nucleosome profile at stress coding regions (Figure 4C, dotted blue line), while it had no effect on Atf1-independent genes (Figure 4D, dotted blue line).

In *S. pombe*, the ATP-dependent chromatin remodellers, Hrp1 and Hrp3, have been described to mediate the formation of a nucleosome array downstream of the TSS, with this correct nucleosome array formation being required for (i) preventing cryptic promoter activity, (ii) silencing antisense transcription and (iii) promoting sense transcription (27,30,31). To investigate whether the lack of Atf1 could mediate a similar effect at stress promoters, we measured the antisense transcription levels in wild-type and *Δatf1* cells at several Atf1-dependent and independent genes, and used cells lacking Hrp1 or Hrp3 as reference strains. Strand-specific reverse-transcription-PCR analysis showed a small but significant increase in antisense transcripts in *Δhrp1* and *Δhrp3* cells at both Atf1-dependent and -independent genes (Figure 4E and F). Strikingly, *Δatf1* cells had an even larger effect than the chromatin remodeller mutants at Atf1-dependent genes, but did not have any effect at independent ones (Figure 4E and F), suggesting an indirect role for Atf1 in preventing cryptic transcription through promoting phased nucleosome arrays. As expected, the sense transcripts decreased at most Atf1-dependent genes and did not change at independent ones (Supplementary Figure S3E and F). Importantly, expression of only the bZIP domain of Atf1 in *Δatf1* cells efficiently prevented antisense transcription at stress coding regions (Supplementary Figure S3G).

## DISCUSSION

Several genome-wide studies have demonstrated that nucleosomes in genomes are often tightly positioned in specific locations. In some organisms, DNA-based computational programs are able to predict nucleosome locations with some success, but increasing evidences have demonstrated that chromatin remodelling activities position nucleosomes around TSSs, as if the +1 nucleosome could serve as a barrier to the +2-to-+*n* nucleosomes in a given gene (for a review, see (29)). However, who locks the +1 nucleosome at its position? Interestingly, nucleosome depleted regions are often preceding the TSSs. Taking advantage of the known nucleosome architecture of stress genes in *S. pombe*, we have here studied the effect of a transcription factor, Atf1, in the establishment of NDRs at promoters and in the nucleation of downstream nucleosome arrays. We show here that in the absence of Atf1 the NDRs are loosely positioned and there is a lack of nucleosome phasing at stress genes.

Fission yeast NDRs seem to be poorly defined by DNA sequence. Thus, while poly(dA-dT) is a strong nucleosome exclusion signal in *S. cerevisiae*, these sequences occur with less frequency in NDRs than elsewhere in the *S. pombe* and human genomes (11,12). Furthermore, when we applied a computational program designed by Segal *et al.* for the prediction of nucleosome positions based on DNA sequence alone to the *S. pombe* genome (6), this program did not reveal NDRs at stress promoters (Supplementary Figure S2), suggesting that *trans* factors and not base composition drive nucleosome exclusion at these Atf1-dependent loci. We present here two types of evidences demonstrating that Atf1 forces the appearance of NDRs at stress promoters: nucleosome scanning experiments in the presence or absence of the TF or its DNA binding site (Figures 1–3), and genome-wide nucleosome maps of wild-type and Δ*atf1* cells (Figure 4).

Using both our genome-wide data and nucleosome scanning of specific Atf1 target genes, we can conclude that binding of the TF to its specific DNA site competes with histones and generates NDRs. In cells lacking Atf1, narrower NDRs are still present at these promoters which may be explained by the fact that stress genes are regulated by multiple TFs (32). Moreover, our results suggest that binding of just a DNA binding domain with high affinity for a specific DNA sequence is enough to exclude nucleosomes from promoters [truncated Atf1 to CRE site (Figure 2D) or DNA binding domain of Gal4 to Gal4 binding site (Figure 3F and G)].

Interestingly, the loss of Atf1 at promoters of stress genes causes an ‘unlocked’ +1 nucleosome, an irregular nucleosome array in coding regions and an increase in antisense transcripts (Figure 4C). Several reports have already shown similar loss of nucleosome phasing downstream of the TSS in chromatin remodeller mutants, and always such nucleosome disorganization brings as a consequence general enhanced cryptic transcription (27,30,31). These mutants, however, did not display changes in the +1 nucleosome positioning conversely to what we report here in the absence of a TF. Probably, chromatin remodellers regulate nucleosome spacing but they do not affect a hypothetical barrier fixing the +1 nucleosome. On the contrary, binding of a TF to DNA may serve as a barrier that in turn allows a correct positioning of downstream nucleosomes but does not affect the regulation of nucleosome spacing *per se*.

RNA polymerase II has also been proposed to influence nucleosome positioning within coding regions in budding yeast (21). However, the effects we describe in our report are only due to the interaction of the TF with its DNA site at promoters, since the bZIP of Atf1, a truncated form of the TF lacking two-thirds of the protein and unable to trigger transcription of target genes after stress imposition (Figure 2A and C), is able to displace histones at promoters (Figure 2D), to promote wild-type nucleosome phasing at stress coding regions (Supplementary Figure S3C) and to suppress enhanced antisense transcription of Δ*atf1* cells (Supplementary Figure S3G).

Our results prompt us to speculate that TFs may function as effective nucleosome barriers to promote proper deposition of the +1 nucleosome. We propose here that Atf1 competes with histones to bind DNA, and functions as an effective barrier that blocks the +1 nucleosome positioning and in turn allows the proper organization of the downstream nucleosome array that is essential for preventing cryptic transcription events. It has been speculated previously that DNA bound factors, such as TF, may function as nucleosome barriers (for a review, see (29)), but to the best of our knowledge ours is the first report demonstrating so *in vivo*. Further studies will be required to demonstrate that the role of Atf1 as a barrier can be extended to other TFs which, by high affinity interaction with their DNA sites, should establish the boundaries towards which nucleosomes are ‘pushed’ by chromatin remodellers.

## SUPPLEMENTARY DATA

Supplementary Data are available at NAR Online.

SUPPLEMENTARY DATA
