# Supplementary material for: Binding of the transcription factor Atf1 to promoters serves as a barrier to phase nucleosome arrays and avoid cryptic transcription
Source: Nucleic Acids Res. 2014 Aug 13;42(16):10351–9. doi: 10.1093/nar/gku704 (PMC4176342; doi:10.1093/nar/gku704)
Supplement: SUPPLEMENTARY DATA [file supp_42_16_10351__index.html]

Binding of the transcription factor Atf1 to promoters serves as a barrier to phase nucleosome arrays and avoid cryptic transcription — SUPPLEMENTARY DATA 

# Binding of the transcription factor Atf1 to promoters serves as a barrier to phase nucleosome arrays and avoid cryptic transcription

## SUPPLEMENTARY DATA

**Files in this Data Supplement:**

- SUPPLEMENTARY DATA
