# Supplementary material for: Binding of the transcription factor Atf1 to promoters serves as a barrier to phase nucleosome arrays and avoid cryptic transcription
Source: Nucleic Acids Res. 2014 Aug 13;42(16):10351–9. doi: 10.1093/nar/gku704 (PMC4176342; doi:10.1093/nar/gku704)
Supplement: SUPPLEMENTARY DATA [file supp_gku704_nar-00605-m-2014-File006.pdf]

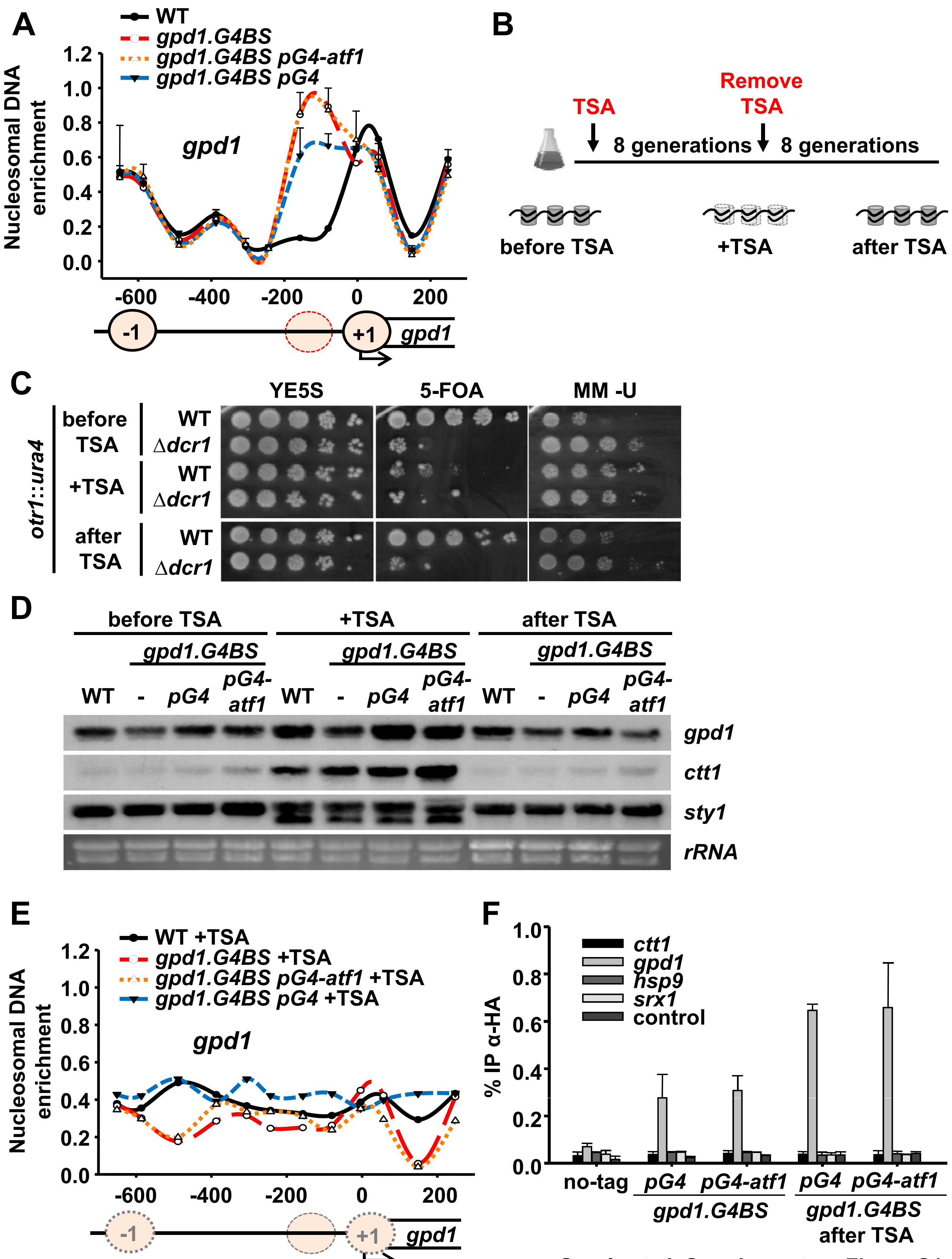

García et al. Supplementary Figure S1

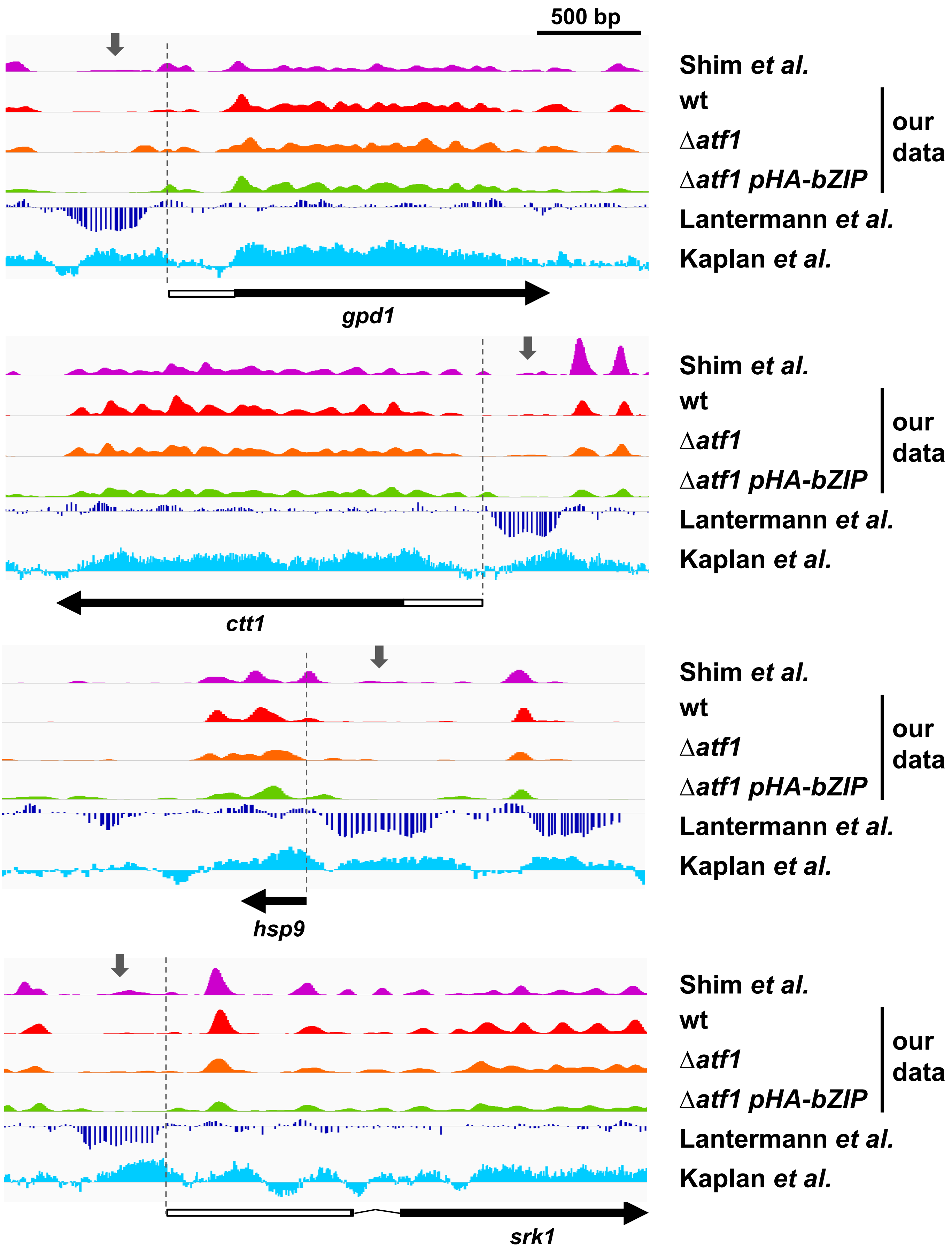

García et al. Supplementary Figure S2

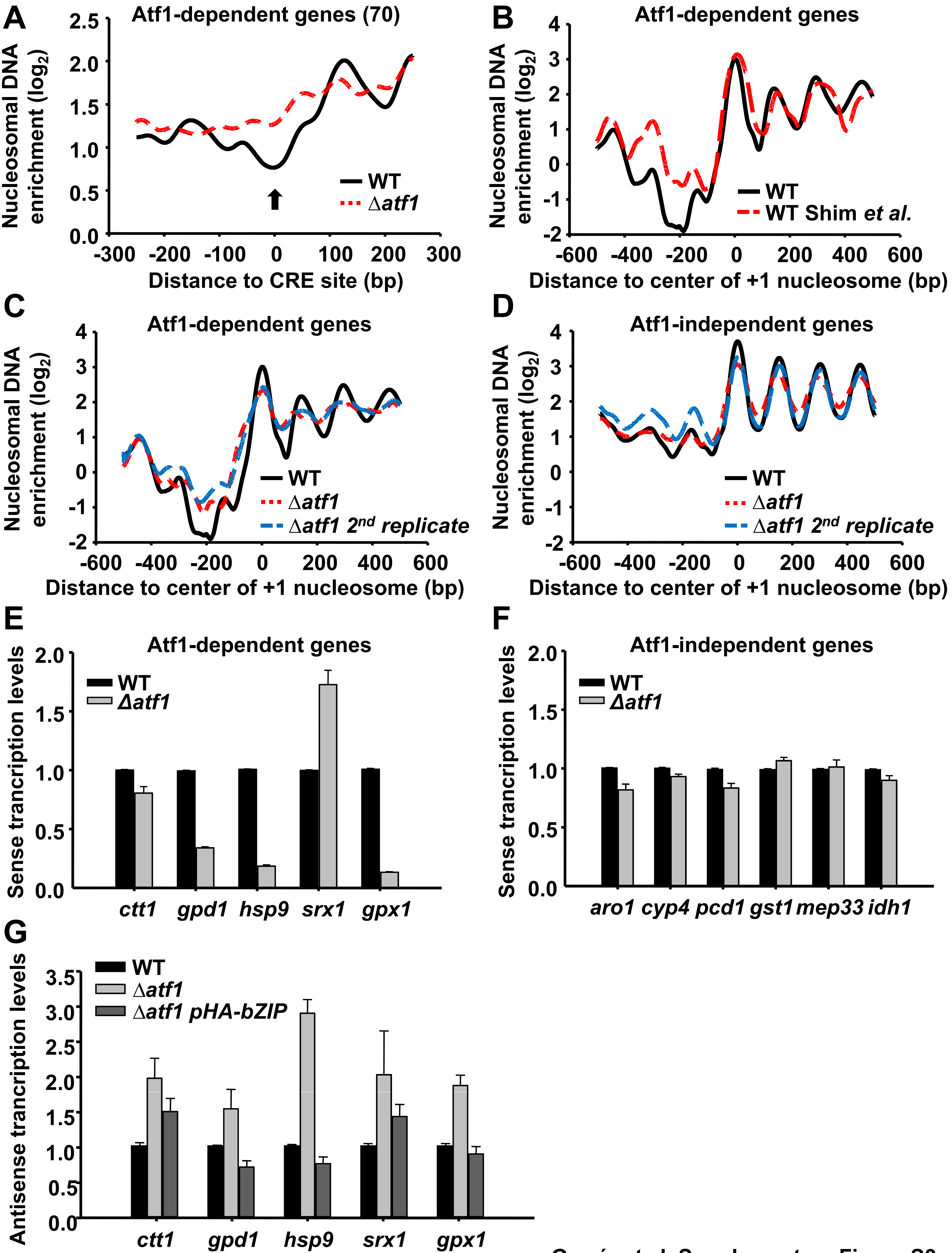

García et al. Supplementary Figure S3

SUPPLEMENTARY FIGURE LEGENDS

Supplementary Figure S1 Trichostatin A (TSA) treatment improves Gal4 and Gal4- Atf1$^{1\text{-}395}$ binding to *gpd1.G4BS* promoter. (**A**) Mono-nucleosomes were isolated from cultures of strains 972 (WT), EP255 (*gpd1.G4BS*), EP286 (*gpd1.G4BS pGal4*) and EP287.ΔbZIP (*gpd1.G4BS pG4-Atf1*), and nucleosome scanning performed as described in Figure 1B. Error bars (SEM) were calculated from biological duplicates. (**B**) Schematic representation of TSA treatment for eight generations to relax chromatin, then the drug was washed out to allow nucleosome repositioning *de novo* for other eight generations. (**C**) TSA treatment alleviates centromeric silencing which is re-established after TSA withdrawal. Cultures of strains DG21 (*otr1::ura4* WT) and DG690 (*otr1::ura4 Δdcr1*) before (before TSA), during (+ TSA), or after (after TSA) TSA withdrawal, were serial diluted and spotted onto rich media plates (YE5S), rich media plates with 5-fluoroorotic acid (5-FOA) or minimal media plates without uracil (MM –U). (**D**) TSA treatment alters expression of the *gpd1, ctt1* and *sty1* genes. RNA from strains as in (A) before, during (+ TSA) and after TSA treatment was analyzed by Northern blot with probes for *gpd1*, *ctt1* and *sty1*. *rRNA* is shown as loading control. (**E**) Nucleosomes are loosely positioned in cells treated with TSA. Mono-nucleosomes were isolated from cultures of strains as in (A) treated with TSA for eight generations. Nucleosome scanning was performed as described in Figure 1B. (**F**) Gal4 binding domain and Gal4-Atf1$^{1\text{-}395}$ physically bind to *gpd1.G4BS*. ChIP analysis of strains 972 (no-tag), EP286 (*gpd1*.G4BS pGal4) and EP287.ΔbZIP (*gpd1*.G4BS pG4-Atf1), before or after TSA treatment, were performed and analyzed as described in Figure 3E.

Supplementary Figure S2 NDRs at stress promoters are not predicted by DNA sequence. View of nucleosome maps at the *gpd1, ctt1, hsp9* and *srk1* genes, comparing data obtained by MNase-seq by Shim *et al*. [ref. (27) in the main text] (pink) or in this study (WT: red, Δ*atf1*: orange, Δ*atf1 pHA-bZIP:* green), by MNase-tilling microarray [ref. (11) in the main text] (dark blue) or predicted by DNA sequence as proposed by Kaplan *et al.* [ref. (6) in the main text] (light blue). NDRs at promoters of these stress genes are indicated with vertical arrows. The transcriptional start sites for each gene according to Lantermann *et al* [ref. (11) in the main text] are indicated with dashed lines.

Supplementary Figure S3 Binding of only the bZIP domain of Atf1 fully restores nucleosome phasing and normal antisense transcription levels at stress genes. (**A**) Composite plot of relative nucleosome occupancy for strains 972 (WT) and MS98 (Δ*atf1*). Seventy stress genes (Supplementary Table S4 and S5) were aligned at their CRE sites and the average of their $\log_2$ nucleosome occupancy was plotted. (**B**) Composite plots of relative nucleosome occupancy for strain WT (solid black line, our data and red dashed line, data from Shim *et al*.,ref. (27) in the main text). (**C**) Composite plots of relative nucleosome occupancy for strains 972 (WT, solid black line) and MS98 (Δ*atf1,* red dashed line and data from second duplicate, blue dashed line; see Materials and Methods). Thirty five stress genes (Supplementary Table S4) were aligned at their +1 nucleosome and the average of their $\log_2$ nucleosomal DNA enrichment data was plotted. (**D**) Composite plot of relative nucleosome occupancy for strains as in (C), but for fifty Atf1-independent genes (Supplementary Table S6). (**E, F**) The expression levels of sense transcripts of Atf1-dependent (*ctt1, gpd1, hsp9, srx1* and *gpx1*; **E**) and -independent (*aro1, cyp4, pcd1, gst1, mep33* and *idh1*; **F**) genes were analyzed by RT-qPCR. (**G**) The expression levels of antisense transcripts of Atf1-dependent (*ctt1, gpd1, hsp9, srx1* and *gpx1*) genes were analyzed by reverse transcription-qPCR. The graphs show the amount of transcripts of each gene relative to that in the WT strain. Error bars (SEM) were calculated from six biological replicates (**E, F**) or triplicates (**G**).

Supplementary Table S1. Genotypes of strains used in this study

Strain	Genotype	Origin
972	*h⁻*	(33)
MS98	*h⁻ atf1::natMX6*	Ref. (14) in main text
EP193	*h⁻ atf1::natMX6 leu1-32*	This work
EP203	*h⁻ atf1::natMX6 leu1-32 psyt1'::HA-atf1::leu1⁺*	This work
EP203.bZIP	*h⁻ atf1::natMX6 leu1-32 psyt1'::HA-atf1^bZIP::leu1⁺*	This work
CH1364	*h⁻ ura5-294 lys7-2 leu1-32*	Ref. (17) in main text
EP184	*h⁺ ctt1'.CRE-to-G4BS leu1-32*	This work
EP213	*h⁺ ctt1'.CRE-to-G4BS leu1-32 psyt1'::HA-Gal4-FLAG::leu1⁺*	This work
EP212.ΔbZIP	*h⁺ ctt1'.CRE-to-G4BS leu1-32 psyt1':: Gal4-FLAG-HA-atf1.ΔbZIP::leu1⁺*	This work
EP255	*h⁺ gpd1'.CRE-to-G4BS leu1-32*	This work
EP286	*h⁺ gpd1.'CRE-to-G4BS leu1-32 psyt1'::HA-Gal4-FLAG::leu1⁺*	This work
EP287.ΔbZIP	*h⁺ gpd1'CRE-to-G4BS leu1-32 psyt1':: Gal4-FLAG-HA-atf1.ΔbZIP::leu1⁺*	This work
EP110	*h⁻ hrp3::kanMX6*	This work
IV69	*h⁻ hrp1::kanMX6*	This work
DG21	*h- otr1R(SphI)::ura4 ura4-DS/E leu1-32 his7-366 ade6-M216*	(34)
DG690	*h- dcr1::kanMX6 otr1R(SphI)::ura4 ura4-DS/E ade6-M210 leu1-32 his7-366*	(34)

Supplementary Table S2. Primers used in nucleosome-scanning assays

Gene	Sequence	Direction	Position from TSS
ctt1	ACTTCCCCAGGAAACACACT	Forward	-495
ctt1	AGCTAGACTTTTCAGTCGCAAA	Reverse	-395
ctt1	GCACAAGTTTGCGACTGAAA	Forward	-426
ctt1	GACACAGACGCTGACATGAAA	Reverse	-328
ctt1	TTTCATGTCAGCGTCTGTGTC	Forward	-349
ctt1	GCCATATCAAATCTCCCAACA	Reverse	-247
ctt1	TAGCGATGGTGTTGGGAGAT	Forward	-277
ctt1	AGCCTATCGTAGTCGCCAAA	Reverse	-181
ctt1 (CRE)	TGGCGACTACGATAGGCTGT	Forward	-199
ctt1 (CRE)	CCGAGATTCCATTGGCTTAG	Reverse	-105
ctt1	GGCTCACTAAGCCAATGGAA	Forward	-131
ctt1	ATTGTCTGGTGTAGAATTACCAACG	Reverse	-39
ctt1	GCCATTTGCGTTGGTAAGAG	Forward	-106
ctt1	TGAAAAGCTGTATATTCAAGCAAC	Reverse	+5
ctt1	TGCTTGAATATACAGCTTTTCACG	Forward	-17
ctt1	TTCGATATTCGAACAAGGGAAT	Reverse	+92
ctt1	GTAATTCCCTTGTTCGAATATCG	Forward	+67
ctt1	GAGGGGAAAAAAAAAGACAGTC	Reverse	+191
ctt1	TTTTTCCCCTCCTAACTCTTCA	Forward	+180
ctt1	GTTGAACGACGAGCAAATGA	Reverse	+281
ctt1	AATCATTTGCTCGTCGTTCA	Forward	+259
ctt1	TCGAGTTCATTTCGCTGATG	Reverse	+370
ctt1	TCAGCGAAATGAACTCGAAA	Forward	+352
ctt1	AGGACCTCCCTTACCCACAC	Reverse	+462
ctt1	GTGGGTAAGGGAGGTCCTGT	Forward	+444
ctt1	ACGACACGTTCAGGAATACG	Reverse	+536
ctt1	CGTCTTTCAACACTTTGATCG	Forward	+491
ctt1	GTAATGTCGTCGGTGCATTC	Reverse	+590
ctt1	TTCCGGTGCTTTTGGTGAAT	Forward	+548
ctt1	CGAGCAACCATAGGGGTTTT	Reverse	+650
ctt1	TTGGTAAGAAAACCCCTATGGT	Forward	+622
ctt1	ACTTTAATGCGAACCCACGA	Reverse	+721
ctt1	CTCGTGGGTTCGCATTAAA	Forward	+700
ctt1	GAATTTAGCGGGATCACGAA	Reverse	+798
gpd1	TCTCTAAGCCGCGTTTTGTT	Forward	-700
gpd1	CTATACCATCTAGGGCCATGC	Reverse	-601
gpd1	TTCTTTAGGAAAGGGCATGG	Forward	-636
gpd1	TCCTTTACATCCTAGCACTCTTT	Reverse	-536
gpd1	GAGTGCTAGGATGTAAAGGA	Forward	-556
gpd1	GCAATGAGAAGGAGAAGCAA	Reverse	-421
gpd1	TTCTCCTTCTCATTGCCCTTA	Forward	-423
gpd1	GATGATAGTTGCGACGAGGA	Reverse	-337
gpd1	CCTCGTCGCAACTATCATCC	Forward	-356
gpd1	TTCGCGATACGCTTTGATAA	Reverse	-255
gpd1	CGCTTCGCAGTTTGTCATTA	Forward	-292

gpd1	TCCGAAGCAAAGAGGAAAAA	Reverse	-194
gpd1	CCTCTTTGCTTCGGACCTTT	Forward	-209
gpd1	ACGCCATAGCAGCCGTACCA	Reverse	-96
gpd1 (CRE)	CTTTGGGTTGACCAAGTGTAG	Forward	-106
gpd1 (CRE)	AAAACACTTCAACGGTGTTGG	Reverse	-31
gpd1	CCAACACCGTTGAAGTGTTT	Forward	-52
gpd1	CGAGACAGATCTATTCCAAGCA	Reverse	+45
gpd1	CTTGTCACGAATCTGCTTGG	Forward	+10
gpd1	ACACTAACCACTCAAAAATGCAG	Reverse	+105
gpd1	GAGTGGTTAGTGTTTGTTGC	Forward	+92
gpd1	CCAGACATTGTGTAGAGAAT	Reverse	+206
gpd1	TGTCTGGATATGGTCAACAAGG	Forward	+199
gpd1	CCAGTTACCGGAGCCAACTA	Reverse	+297
gpd1	GGTGTAGTTGGCTCCGGTAA	Forward	+273
gpd1	ACATGCGGACCTTACTTCTGA	Reverse	+373
gpd1	TGTGGGTCTTTGAGGAGGAG	Forward	+370
gpd1	TGCCGGGTAAGTATTTGACA	Reverse	+469
gpd1	CAAATACTTACCCGGCATCG	Forward	+452
gpd1	GGAACGACAAAGACAAGGATG	Reverse	+551
gpd1	TAGACGTGCCGACATCCTT	Forward	+518
gpd1	GAAATACCAACGGCACCAG	Reverse	+620
gpd1	GTCTCATTCGCCCTGGTG	Forward	+589
gpd1	GTTTCTCGCTGATAACCTCAGA	Reverse	+688
srx1 (CRE)	TTTCGCTAACATGCTGACAA	Forward	-146
srx1 (CRE)	GCCCCACAACCTATCTTATCC	Reverse	-29
hsp9 (CRE)	CCTTGGATCGCAGTTTTCTC	Forward	-174
hsp9(CRE)	CTTGTCCAATTGCTCATGTCA	Reverse	-81
mei2(control)	CCAAAGAAGCAGCATTCAAA	Forward	+2527
mei2(control)	GAACAGAACCTCCCTGCATA	Reverse	+2634

Supplementary Table S3. Primers used in strand-specific RT-qPCR

Gene	Sequence	Direction	Position from ATG	Position from TSS
ctt1	CTCGTGTGGGTAAGGGAGGT	Forward	+80	+440
ctt1	ATGGACGACACGTTCAGGA	Reverse	+180	+540
gpd1	GCTCCGGTAACTGGGGTACT	Forward	+87	+285
gpd1	CTCCTCAAAGACCCACATGC	Reverse	+189	+387
hsp9	CAAGGCCAAGGAATCCATTA	Forward	+81	+81
hsp9	GAGCCTTGTCATGAGCCTCT	Reverse	+181	+181
srx1	TTCATGCGGTTTGACTTCAG	Forward	+141	+288
srx1	CCCCCAAAGGCAAAATAATA	Reverse	+245	+392
gpx1	CGACTTGGCTCCTAAGGACA	Forward	+15	+15
gpx1	AATCCACATTTGCTCGCTGT	Reverse	+113	+113
aro1	TTAAAGATGCCAAGGCTGAAG	Forward	+194	+194
aro1	CAATCTTCAATTTCCGCTTTC	Reverse	+290	+290
cyp4	CGGAGAAAAGGGATTTGGAT	Forward	+192	+222
cyp4	CCAGTACCATCACCCTTCGT	Reverse	+290	+320
pcd1	AGCAGTCAATCGCACCATGT	Forward	+169	+299
pcd1	CCATGGTCAAATCTCCGTTC	Reverse	+265	+395
gst1	ATCTTTGCCTCGTGACCATC	Forward	+264	+264
gst1	CTAAACCATCCAGCCTGTCC	Reverse	+365	+365
mep33	CGGTCTACCAAGGTTCAAGC	Forward	+109	+453
mep33	GTTTGCGAAGAGCTTCCTGA	Reverse	+205	+554
idh1	AAATCCTCAGCTTTTCAACCA	Forward	+22	+82
idh1	CTGTCACTGCATTGCTCGTT	Reverse	+121	+181

Supplementary Table S4. List of the top 35 genes up-regulated in response to 0.5 mM H_2O_2 (35)

Gene names	Chromosome	Strand	ORF left position	ORF right position	nucleosome +1	CRE site
SPBC660.05	II	+	202536	202967	202302	202209
SPAC23H3.15c:SPAC25H1.01c	I	-	2523629	2524606	2524880	2524932
SPBC24C6.09c	II	-	2335262	2337739	2337715	2337818
SPAC4H3.03c	I	-	3826640	3828589	3829160	3829187
pyp2:SPAC19D5.01	I	+	5202823	5204958	5202100	5201878
SPCC757.03c	III	-	47741	48475	48783	48644
ish1:SPBC365.12c	II	-	2516249	2518303	2518785	258840
SPAC2H10.01	I	+	5274171	5275613	5273050	5274171
SPAC22F8.05	I	+	4792308	4794983	4791890	4791800
SPBC725.03	II	+	1207040	1207813	1207100	1206971
oca2:SPCC1020.10	III	-	758901	760853	762700	762814
srx1:SPBC106.02c	II	-	376462	376836	376800	377115
SPAC23G3.13c:SPAC22H12.01c:mug35	I	-	893719	894423	894475	895018
SPBC725.10	II	+	1224974	1225462	1224920	1224499
ctt1:cta1:SPCC757.07c	III	-	56927	58465	58665	58893
SPCC417.13:SPCC191.01	III	+	1706461	1706997	1705900	1705993
rsv2:SPBC1105.14	II	+	3532931	3534844	3533050	3532881
SPCP31B10.06:mug190	III	+	533322	536888	532620	532574
SPBC1105.13c	II	-	3531760	3532188	3532530	3532889
SPAC32A11.02c	I	-	2447948	2450503	2451032	2451064
SPBC11C11.06c	II	-	3359360	3359896	3359940	3359938
SPBC21C3.19	II	+	3830167	3830487	3830083	3829924
srk1:mkp1:SPCC1322.08	III	+	1302900	1304865	1302200	1301880
SPAC16A10.01	I	+	3077676	3080168	3080055	3077622
SPAC57A7.02c:SPAC167.06c:mug143	I	+	1550133	1550498	1550118	1550036
SPCC338.12	III	-	1355276	1355509	1355640	1355689
ssa1:SPAC13G7.02c	I	-	2295819	2297753	2297750	2298314
SPBC428.10	II	+	461306	463561	460945	460733
SPCC4G3.03	III	-	466796	467839	467860	468099
SPAC57A7.05	I	-	1534588	1538601	1540125	1540610
hsp16:SPBC3E7.02c	II	-	2660490	2660921	2661130	2661060
SPCC16A11.15c	III	-	896182	896535	896915	897141
cgs1:SPAC8C9.03	I	+	3643239	3644742	3643140	3642965
ntp1:SPBC660.07	II	+	207078	209285	2069970	206512
SPBC16D10.08c	II	-	3612359	3615076	3615254	3615709

Supplementary Table S5. List of aditional 35 genes with CRE site (36)

Gene names	Chromosome	Strand	ORF left position	ORF right position	CRE site
SPCC320.03	III	-	168525	165922	170909
SPBC1685.13	II	+	526091	526642	525443
SPBC32F12.11:tdh1	II	+	2807637	2808647	2807395
SPCC1672.02c:sap1	III	-	562353	563117	563843
SPAP8A3.04c:hsp9	I	-	5320399	5320605	5320739
SPACUNK4.15	I	-	2881105	2881731	2882242
SPBC215.05:gpd1	II	+	4035814	4036971	4035445
SPCC576.03c:tpx1	III	-	2085036	2085614	2085824
SPBC530.10c:anc1	II	-	811413	812381	812990
SPBC725.02:mpr1	II	+	1203757	1204644	1202995
SPCC736.15	III	+	349172	350227	348400
SPBC660.16	II	+	230368	233061	230260
SPBC19C2.07:fba1	II	+	1688367	1689443	1688177
SPCC576.17c	III	-	2111638	2113390	2113775
SPCC553.10	III	-	283422	284471	285345
SPCC622.12c	III	-	1419457	1420812	1421221
SPAC25B8.12c	I	-	4177021	4177932	4178329
SPAC343.12:rds1	I	+	1668071	1669279	1667637
SPBC4B4.08:ght2	II	+	3427572	3429167	3427029
SPAC26F1.07	I	-	5170883	5171848	5172152
SPAC27E2.11c	I	-	4009573	4009818	4010000
SPBC14F5.04c:pgk1	II	-	4158172	4159416	4159697
SPAC9E9.09c	I	-	4453560	4455071	4455851
SPCC13B11.01:adh1	III	+	1591359	1592411	1590984
SPCC825.03c:psy1	III	-	1028292	1029146	1029369
SPAC9E9.01	I	+	4434861	4435201	4434770
SPACUNK4.17	I	-	2873032	2874252	2874728
SPBC32F12.03c:gpx1	II	-	2789071	2789547	2789652
SPBC16H5.02:pfk1	II	-	2297466	2300294	2300620
SPAC26H5.09c	I	-	4137573	4138682	4139341
SPBC3H7.02	II	-	1884365	1886998	1887836
SPCC1235.01	III	+	174183	176159	173785
SPBC19C7.04c	II	-	2825692	2826066	2826838
SPAC1751.01c	I	-	378192	380354	381580
SPBC17G9.11c:pyr1	II	-	2190874	2194431	2194609

Supplementary Table S6. List of 50 genes non-regulated in response to 0.5 mM H_2O_2 (35)

Gene names	Chromosome	Strand	ORF left position	ORF right position	nucleosome +1
aro1:SPAC1834.02	I	+	4693771	4698492	4.694.012
cyp4:SPBP8B7.25	III	+	3685901	3686599	3.685.972
SPCC4B3.16	III	-	1146383	1147141	1.147.027
SPAC630.09c	I	-	362769	363602	363.537
SPAC17G8.05	I	+	2350327	2351019	2.350.492
SPBC1198.05	II	+	181695	182303	181.707
pcd1:SPAC6G9.05	I	+	3253513	3254370	3.801.542
gst1:SPCC191.09c	III	-	1719325	1720014	1.720.052
mep33:SPBC28F2.02	II	+	1574527	1575405	1.574.392
idh1:SPAC11G7.03	I	+	3966835	3967905	3.966.831
SPAC1A6.03c	I	-	1070061	1072127	1.072.330
SPBC4C3.06	II	-	3124204	3126701	3.126.671
SPCC1442.05c	III	-	1776594	1777127	1.777.123
SPAC56F8.07	I	+	1138566	1139117	1.138.723
SPBC1685.04	II	+	502873	503850	503.973
SPAP14E8.04	I	+	1926739	1927752	1.926.777
ppi1:SPBC28F2.03	II	+	1576329	1576817	1.576.425
aps2:SPBC685.04c	II	-	2776485	2776916	2.777.152
rpc25:SPBC2G5.07c	II	-	2592258	2593170	2.593.362
SPAC977.08	I	+	44704	45414	44.798
btb3:SPAC13D6.04c	I	-	2251500	2253189	2.253.152
cdc17:SPAC20G8.01	I	+	1387944	1390352	1.388.024
SPAC10F6.13c	I	-	1230788	1232017	1.232.011
SPBC13A2.01c	II	-	3399336	3400073	3.400.067
SPAC8F11.09c	I	-	2868517	2869377	2.869.391
SPCC63.06	III	+	845924	846919	846.027
rga7:SPBC23G7.08c	II	-	2110807	2112894	2.112.873
btb2:SPBC25B2.06c	II	-	2607314	2608370	2.608.399
trk1:SPAC3F10.02c	I	-	2818256	2820781	2.821.073
bgs1:SPBC19G7.05c	II	-	2351054	2356243	2.357.041
nfs1:SPBC21D10.11c	II	+	2422760	2424265	2.422.913
SPBC19C2.10	II	+	1695221	1696841	1.695.336
orb6:SPAC821.12	I	+	1008439	1010160	1.008.652
sub1:SPAC16A10.02	I	+	3081012	3081472	3.084.073
cog1:SPAC144.15c	I	-	4683276	4685381	4.685.340
SPAC7D4.03c	I	+	2634211	2636871	2.634.319
meu27:SPCC1259.14c	III	-	1061985	1064195	1.064.240
rpl302:SPAPB8E5.06c	I	-	4917825	4918991	4.918.997
SPCC4G3.13c	III	+	445404	446051	445.500
pep3:SPCC790.02	III	+	2242505	2245354	2.242.592
rps2802:SPCC285.15c	III	-	1827104	1827360	1.827.374
cys2:SPBC106.17c	II	-	411120	412634	412.666
cox9:SPCC1259.05c	III	-	1042639	1042877	1.042.896
SPAC3A11.01	I	-	3471020	3474807	3.474.815
rpn502:SPAPB8E5.02c	I	-	4909497	4910875	4.910.706
exo70:SPBC106.20	II	+	417319	419476	419.148
SPCC285.03	III	+	1797992	1799806	1.798.235
ptp4:SPBC3D6.05	II	+	1278358	1279373	1.278.462
rud3:SPBC119.12	II	+	740275	741480	740.373
SPBC1734.03	II	+	1064200	1066401	1.064.410

SUPPLEMENTARY REFERENCES

33. Leupold, U. (1970) Genetical methods for *Schizosaccharomyces pombe*. *Methods Cell Physiol.*, **4**, 169-177.
34. Li, F., Goto, D.B., Zaratiegui, M., Tang, X., Martienssen, R. and Cande, W.Z. (2005) Two novel proteins, dos1 and dos2, interact with rik1 to regulate heterochromatic RNA interference and histone modification. *Curr Biol*, **15**, 1448-1457.
35. Chen, D., Wilkinson, C.R., Watt, S., Penkett, C.J., Toone, W.M., Jones, N. and Bahler, J. (2008) Multiple pathways differentially regulate global oxidative stress responses in fission yeast. *Mol Biol Cell*, **19**, 308-317.
36. Eshaghi, M., Lee, J.H., Zhu, L., Poon, S.Y., Li, J., Cho, K.H., Chu, Z., Karuturi, R.K. and Liu, J. (2010) Genomic binding profiling of the fission yeast stress-activated MAPK Sty1 and the bZIP transcriptional activator Atf1 in response to H2O2. *PLoS One*, **5**, e11620.
